# Editorial: Brown and beige adipocytes: from molecular mechanisms to therapeutic potentials

**DOI:** 10.3389/fendo.2026.1797700

**Published:** 2026-02-12

**Authors:** Xinrui Li, Sharmeen Islam, Min Du

**Affiliations:** Nutrigenomics and Growth Biology Laboratory, Department of Animal Sciences, Washington State University, Pullman, WA, United States

**Keywords:** brown and beige adipocytes, environment, metabolic syndrome, mitochondria, thermogenesis

## Introduction

Brown and beige adipocytes are key regulators of systemic energy balance and metabolic homeostasis through their capacity for adaptive thermogenesis and metabolic remodeling. In contrast to energy-storing white adipocytes, brown and beige adipocytes dissipate energy as heat and contribute to whole-body energy expenditure. Impairment of thermogenic adipocyte function has been increasingly linked to obesity, insulin resistance, metabolic syndrome, and related cardiometabolic disorders (1). Beyond thermogenesis, these cells exhibit remarkable plasticity, allowing them to adapt dynamically to inflammatory, environmental, nutritional, and endocrine cues (2). This Research Topic brings together five compelling studies that advance our understanding of the molecular mechanisms governing thermogenic adipocyte function and their responses to diverse environmental, pharmacological, and pathological stimuli.

## Environmental regulation of thermogenic adipocytes

Environmental temperature represents a fundamental regulator of adipose tissue biology and metabolic homeostasis. The contribution by Paz et al. examined how short-term housing temperature alterations affected metabolic parameters and adipose tissue remodeling in female mice. Their findings showed that brief cold exposure reduced adipocyte size in inguinal white adipose tissue and increased vascularization in brown adipose tissue, accompanied by proteomic changes in pathways related to mitochondrial function and mTOR signaling, without persistent effects on systemic metabolism.

Extending this environmental perspective, Yi et al. leveraged single-nucleus RNA sequencing to delineate the temporal remodeling of the adipose tissue microenvironment following cold exposure. This high-resolution profiling resolved the cellular heterogeneity within distinct adipose depots and identified specific adipocyte, stromal, and immune cell subsets that collectively governed thermogenic remodeling and, in subcutaneous depots, beige adipocyte recruitment. The emergent metabolic signatures across these cell populations provided insight into the orchestrated, multicellular response that underlays thermogenic activation. Such integrative mapping of adipose tissue niches refined current understanding of how intercellular communication networks regulate browning, UCP1-independent thermogenic programs, and broader metabolic adaptation.

## Pharmacological modulation of adipocyte remodeling

The potential for pharmacological strategies to induce favorable adipose tissue remodeling attracted considerable attention in the context of metabolic disease. Okla et al. interrogated the actions of apigenin, a naturally occurring dietary flavonoid, on peripheral and skeletal adipocyte remodeling under conditions of β3-adrenergic receptor (β3-AR) stimulation and Toll-like receptor 4 (TLR4)–dependent inflammatory activation. In doing so, this work integrated nutritional biochemistry with adipose tissue cell biology to illustrate how diet-derived bioactive molecules recalibrated signaling networks that governed thermogenic activation and adipocyte plasticity. The concurrent targeting of β3 AR and TLR4 pathways was particularly innovative, as it encompassed both the canonical sympathetic thermogenic axis and the immune–metabolic circuitry that constrained or facilitated adipocyte function. Collectively, these findings supported the concept that apigenin and related flavonoids might potentiate thermogenic capacity and adipose tissue remodeling through convergent, mechanistically complementary pathways, thereby nominating this class of compounds as promising candidates for adjunctive metabolic therapies.

## Pathological disruption of BAT function

While much research focused on augmenting thermogenic adipocyte activity, delineating conditions that compromised BAT function are equally critical. Ji et al. investigated how chronic intermittent hypoxia, a defining feature of obstructive sleep apnea (OSA), perturbed BAT, with particular emphasis on microRNA-mediated regulatory mechanisms. Using differential expression profiling and integrative bioinformatics analysis, the study identified hypoxia-responsive microRNAs in BAT; through target prediction and pathway enrichment analyses, it predicted their regulatory effects on genes governing mitochondrial function, lipid metabolism and thermogenic activation. Rather than fully establishing a causal axis, these data provided mechanistic support for the concept that BAT dysfunction contributed to the heightened metabolic disease risk associated with OSA. The delineation of hypoxia-associated microRNA signatures in BAT, in turn, pointed to testable candidates for future biomarker development and targeted therapeutic modulation, pending functional validation in preclinical and clinical settings.

## Environmental pollutants and adipose tissue dysfunction

The contribution by Lv et al. delineated the metabolic consequences of chronic exposure to bisphenol AF (BPAF), an environmental endocrine-disrupting compound used as a bisphenol A substitute. This study demonstrated that prolonged BPAF administration differentially exacerbated fat deposition in mice maintained on normal chow versus high-fat diets, in part through dysregulation of hepatic and adipose lipid metabolism and adipocyte hypertrophy–driven expansion of white adipose depots. The documented dose regimen and clear diet-dependent divergence in adipose and hepatic responses underscored the complex interplay between environmental toxicants, nutritional status, and adipose tissue function. Notably, BPAF exposure was associated with reduced resting body temperature and suppression of thermogenic regulators in brown adipose tissue, indicative of impaired thermogenic capacity and a shift in the balance between energy storage and expenditure that may have amplified obesogenic risk. Against the backdrop of widespread human exposure to bisphenol analogs, these findings raised important public health concerns and positioned endocrine-disrupting chemicals as salient modifiers of adipose tissue biology and systemic metabolic homeostasis.

## Synthesis and perspective

These five studies delineated thermogenic adipocytes as a highly dynamic metabolic cell population whose identity and function are continuously shaped by external stimuli and local tissue context. Rather than acting as isolated heat-producing units, thermogenic adipocytes function as integrative hubs that translate environmental cues, immune signals, and intracellular regulatory programs into adaptive or maladaptive metabolic outcomes (3). Together, this body of work emphasized that thermogenic capacity represents a reversible cellular state, maintained through coordinated regulation across signaling, transcriptional, and epigenetic layers.

A central theme emerging from these studies is that thermogenic regulation is inherently multicellular and context dependent. Crosstalk between adipocytes and immune, stromal, and neural components within the adipose niche influences browning efficiency and the sustainability of thermogenic programs, while intrinsic regulators such as microRNAs and metabolic checkpoints modulate cellular responsiveness (4). Together, these observations highlighted thermogenic adipocyte function as an emergent property of coordinated intercellular interactions and cell-intrinsic regulatory networks within adipose tissue.

Overall, this Research Topic reframes thermogenic adipocytes as context-sensitive regulators of metabolic homeostasis rather than static energy-dissipating cells. By elucidating how thermogenic programs are shaped by environmental inputs, intercellular interactions, and intrinsic regulatory circuits, this Research Topic advances a more integrated understanding of adipose tissue plasticity with clear relevance to metabolic disease.
